# Medial meniscus extrusion and varus tilt of joint line convergence angle increase stress in the medial compartment of the knee joint in the knee extension position -finite element analysis-

**DOI:** 10.1186/s40634-022-00490-y

**Published:** 2022-05-27

**Authors:** Takuhei Kozaki, Daisuke Fukui, Ei Yamamoto, Daisuke Nishiyama, Manabu Yamanaka, Akimasa Murata, Hiroshi Yamada

**Affiliations:** 1 Department of Orthppaedics Surgery, Wakayama Medical University, Wakayama, Japan; 2 Department of Biomedical Engineering, Faculty of Biology-Oriented Science and Technology, Kindai University, Wakayama, Japan

**Keywords:** Medial meniscal extrusion, Joint line convergence angle, Stress load, Finite element analysis

## Abstract

**Purpose:**

Although it has been recognized that the medial meniscus extrusion (MME) leads to progressive cartilage loss and osteoarthritis (OA), about 20% of cases with MME had minor symptoms and poor progression of knee OA. However, it is still unclear which patients will have minimal symptoms or will not progress to degeneration. The purpose of this study is to compare the effect of the relationship between the MME and Joint line convergence angle (JLCA) on knee stress with the finite element (FE) analysis method.

**Methods:**

The 65 year-old female was taken computer tomography (CT) from thigh to ankle. A 3-dimentional nonlinear FE model was constructed from the patient’s DICOM data. We made the six models, which was different from JLCA and MME. Contact stresses on the surfaces between femoral and tibial cartilages and both side of meniscus are analyzed.

**Results:**

As the JLCA or MME increased, the stress load on the medial compartment increased. The effect of MME was stronger on the femoral side, while the effect of JLCA was stronger for the tibia and meniscus. If the JLCA was tilted valgus, the stress in the medial compartment did not increase even in the presence of MME.

**Conclusions:**

This study revealed that the MME is associated with increased a stress loading on medial compartment structures. Furthermore, this change was enhanced by the varus tilt of the JLCA. In the case of valgus alignment, the contact pressure of the medial compartment did not increase so much even if with the MME.

**Level of evidence:**

Level V

## Introduction

Knee osteoarthritis (OA), one of the most common diseases encountered in orthopedic surgery, leads to considerable medical and social problems [12]. Although knee OA is regarded as a whole joint disease with a multifactorial etiology, meniscal degeneration is strongly associated with knee OA.

It has been recognized that medial meniscus extrusion (MME) leads to progressive cartilage loss, OA, and spontaneous osteonecrosis of the knee (SONK) [3, 23]. MME is associated with severe meniscal degeneration and medial meniscus posterior root tear (MMPRT) [4]. From previous report, it is implicated that MME greatly increases the contact pressure applied to the knee joint and have a significant effect on cartilage and bone tissue [20]. Therefore, pull-out repair and/or centralization for MME and MMPRT are commonly performed for the surgical repair of the medial meniscus [1, 14]. Medium-term results of pull-out repair have been reported in recent years, showing that the clinical scores have improved and fewer patients have undergone total knee arthroplasty than those that have undergone conservative treatment [6]. However, Lerer reported that even with MME and MMPRT, approximately 20% of cases had minor symptoms and poor progression of knee OA [15]. Such patients may not require surgical treatment, but it is still unclear which patients will have minimal symptoms or will not progress to degeneration. Willinger et al. reported that total medial meniscal resection and varus alignment increase stress on the medial knee compartment by approximately 210%, while valgus alignment only increases contact pressure by approximately 20%, even after total meniscal resection in a cadaveric knee biomechanical study [22]. Based on this report, we hypothesized that even if there is MME, the contact stress on the knee joint does not always increase significantly, depending on the alignment. However, the extent to which differences in alignment affect the stress load on the knee with MME is still unclear. The purpose of this study was to compare the effect of the relationship between MME and knee alignment on knee stress using the finite element (FE) analysis method.

## Methods

### FE model

**Fig. 1:**
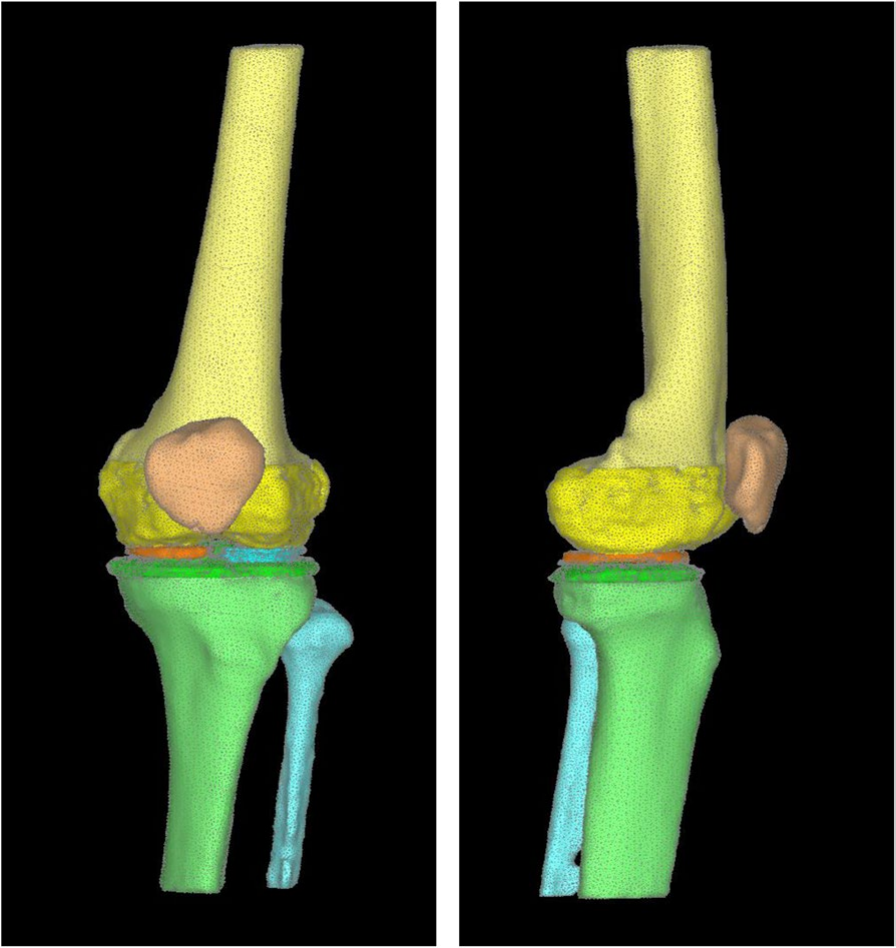
Finite element model. Finite element model from femur to tibia and fibula, which was based on a 65-year-old woman. The KL classification was grade 1, the FTA was 177.6°, the MPTA was 86.3°, the LDFA was 83°, and the JLCA was 1.1° in the full leg weight-bearing radiographs. MRI showed no posterior root tear of the medial meniscus, and the MME was 1.2 mm at the middle of the anteroposterior diameter of the tibia. KL; Kellgren-Lawrence, FTA: femolo-tibial angle, MPTA; medial proximal tibial angle, LDFA; lateral distal femur angle, JLCA; joint line convergence angle, MME; medial meniscus extrusion

**Fig. 2:**
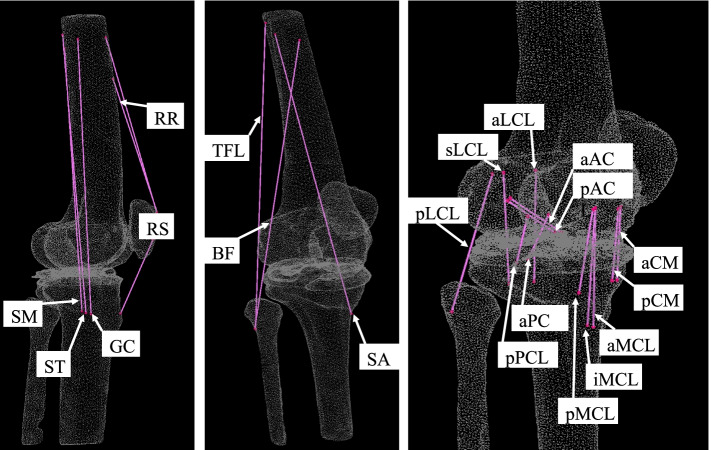
Finite element model with each of the ligaments and muscles. FE models, which were constructed from the left femur, patella, tibia, and fibula, were equipped with triangular shell elements (thickness, 2 mm; size, 3 mm) for the outer surface of the cortical bone and tetrahedral solid elements with a size of 2 mm. Ligaments and soft tissues around the knee joint were used in accordance with previous reports. RS; Rectus femoris (straight head), RR; Rectus femoris (reflected head), TFL; Tensor fasciae latae, SA; Sartorius, SM; Semimembranosus, ST; Semitendinosus, BF; Biceps femoris, GC; Gracilis, ACL; anterior cruciate ligament, PCL; posterior cruciate ligament, LCL; lateral cruciate ligament, MCL; medial cruciate ligament, MC; medial capsule

This study was approved by the institutional ethics committee. The participants provided written informed consent. A 65-year-old female patient with left knee pain underwent computed tomography (CT) from the thigh to the ankle. During the CT simultaneous scanning of a calibration phantom (BMAS 200; Kyoto Kagaku, Kyoto, Japan) containing hydroxyapatite rods was performed to determine bone density. The Kellgren-Lawrence (KL) classification was grade 1, the femolo-tibial angle (FTA) was 177.6°, the medial proximal tibial angle (MPTA) was 86.3°, the lateral distal femur angle (LDFA) was 83°, and the joint line convergence angle (JLCA) was 1.1° in the full leg weight-bearing radiographs. MRI showed no posterior root tear of the medial meniscus, and the MME was 1.2 mm at the middle of the anteroposterior diameter of the tibia. A 3-dimensional nonlinear FE model was constructed from the patient’s Digital Imaging and Communication in Medicine (DICOM) data and analyzed with the Mechanical Finder® version 10 software (RCCM, Tokyo, Japan). FE models, which were constructed from the left femur, patella, tibia, and fibula, were equipped with triangular shell elements (thickness, 2 mm; size, 3 mm) for the outer surface of the cortical bone and tetrahedral solid elements with a size of 2 mm. Ligaments and soft tissues around the knee joint were used in accordance with previous reports [21] (Figs. 1 and 2). We created six models varying in knee joint alignment and MME to evaluate the influence of JLCA and extrusion of meniscus.

### MME

Because the criterion for MME was defined as a distance of 3 mm or more between the peripheral border of the meniscus and the edge of the tibial plateau [15], the amount of MME was set at 3 mm.

### JLCA

Although a standard value for JLCA, or the angle made by a tangential line between the femoral condyles and the tibial plateau, is not clearly defined, Nakano et al. examined the coronal alignment of normal knees in adults and reported that the range of JLCA was 1.1 ± 1.7 in middle aged men and 1.0 ± 1.5 in middle women (mean ± SD) [16]. Based on this report, JLCA by 2 degrees in the varus and valgus directions, respectively.

### Group

The two initials of the first half of the group name were used as VA in which the JLCA was tilted in the direction to varus with two degrees, VG was changed to valgus direction with two degrees, and NT was unchanged from the original data. The latter two initials of the group name were EX for the group that extruded the medial meniscus by 3 mm from the original data and NE for the group that did not. These were combined to create a total of six groups (Table 1)

**Table 1:** The six models, varying in joint line convergence angle of knee joint and medial meniscus extrusion

	**Valgus**	**Neutral**	**Varus**
without extrusion	VGEX	NTEX	VAEX
with extrusion	VGNE	NTNE	VANE

### FE analysis

A load of 560 N was applied axially to the femoral bone, specifically to the intermittent condyle of the knee joint. The sides of both the distal tibia and fibula were completely restrained. The material properties of the muscles and ligaments of the knee joint are listed in Table 2 [9, 17, 19]. The Young’s moduli and Poisson’s ratios for the femur, patella, tibia, and fibula had previously been reported, and these were calculated for this research using the equations proposed [13]. Contact analysis was performed on the surface between the femoral cartilage and both sides of the meniscus. It was also recognized on the surface between the femoral cartilage and tibial cartilage. FE analysis was performed to measure the average stress in the medial and lateral condylar cartilage of the knee joint. The von Mises stress and angular motion of the hip joint were analyzed in each model.

**Table 2:** Properties of the muscles and ligaments

**Materials**	**k(N/mm)**
Rectus femoris (straight head)	39
Rectus femoris (reflected head)	39
Tensor fasciae latae	13
Sartorius	92
Semitendinosus	44
Semimembranosus	100
Biceps femoris	74
Gracilis	28
aACL	5000
pACL	5000
aPCL	9000
pPCL	9000
aLCL	2000
sLCL	2000
pLCL	2000
aMCL	2750
iMCL	2750
pMCL	2750
aMC	1000
pMC	1000

Ligaments and soft tissues around the knee joint were constructed based on past study [14]

*ACL *anterior cruciate ligament, *PCL *posterior cruciate ligament, *LCL* lateral cruciate ligament, *MCL* medial cruciate ligament, *MC* medial capsule

## Results

The average von Mises stress on the medial and lateral condylar cartilage of the tibia and femur side and meniscus were shown in Table 3.

**Table 3:**
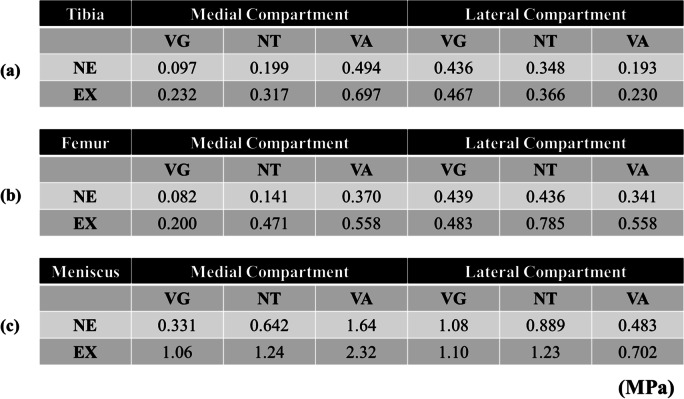
Von mises stress on each compartment of the knee joint

The von Mises stress of medial compartment on tibia (a), femur (b), and meniscus (c), respectively increased with the varus and extrusion of the medial meniscus. *NE *non-extrusion of medial meniscus, *EX *extrusion of medial meniscus, *VG *valgus, *NT *neutral, *VA *varus

### Condylar cartilage on the tibia side (Fig. 3)

**Fig. 3:**
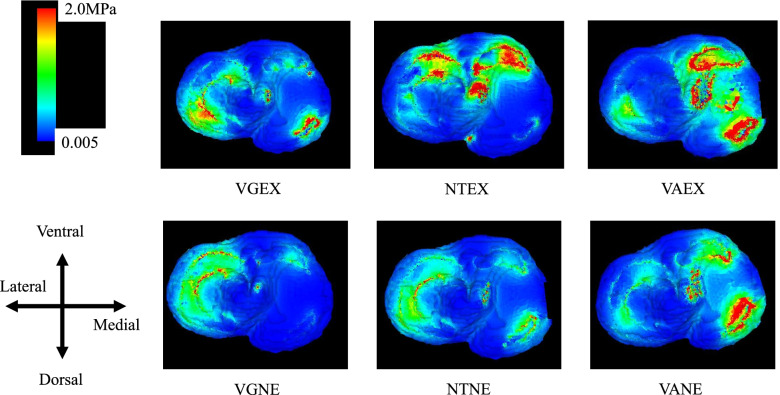
Axial view of the knee cartilage on the tibial side showing von Mises stress. The von Mises stress was most concentrated in the medial cartilage on the tibial side in the VAEX models, followed by that in the VANE model. VGNE; valgus and non-extrusion of medial meniscus, VGEX; valgus and extrusion of medial meniscus; NTNE; neutral and non-extrusion of medial meniscus, NTEX; neutral and extrusion of medial meniscus, VANE; varus and non-extrusion of medial meniscus, VAEX; varus and extrusion of medial meniscus

#### NTNE versus VANE and VGNE

The average stress on the medial condylar cartilage for VANE increased by 2.49-fold compared with that for NTNE. On the other hand, for VGNE it increased by 0.49-fold.

The average stress on the lateral condylar cartilage for VANE increased by 0.49-fold. On the other hand, for VGNE it increased by 1.25-fold.

#### NTNE versus NTEX, VAEX, and VGEX

The average stress on the medial condylar cartilage for NTEX increased by 1.60-fold, and that for VAEX increased by 3.52-fold; however, the stress for VGEX increased by 1.17-fold.

The average stress on the lateral condylar cartilage for NTEX was not raised as much, with an increase of 1.05-fold, and VAEX by 0.66-fold, but VGEX increased by 2.36-fold.

### Condylar cartilage on the femoral side (Fig. 4)

**Fig. 4:**
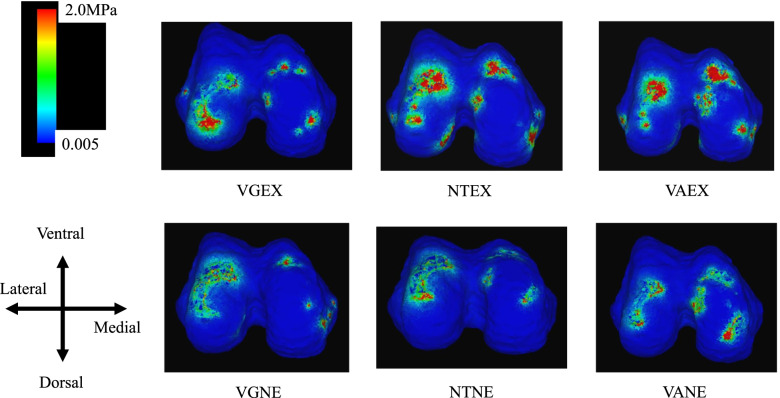
Axial view of the knee cartilage on the femoral side showing von Mises stress. The von Mises stress was most increased in the medial cartilage on the femur side in the VAEX model, followed by the NTEX model. VGNE; valgus and non-extrusion of medial meniscus, VGEX; valgus and extrusion of medial meniscus; NTNE; neutral and non-extrusion of medial meniscus, NTEX; neutral and extrusion of medial meniscus, VANE; varus and non-extrusion of medial meniscus, VAEX; varus and extrusion of medial meniscus

#### NTNE versus VANE and VGNE

The average stress on the medial condylar cartilage for VANE increased by 2.62-fold. On the other hand, for VGNE this increased by 0.58-fold. The average stress on the lateral condylar cartilage for VANE increased by 0.78-fold. On the other hand, for VGNE this did not increase 1.00-fold.

#### NTNE versus NTEX, VAEX, and VGEX

The average stress on the medial condylar cartilage for NTEX increased by 3.34-fold, and that for VAEX increased by 3.96-fold, but that for VGEX increased by 1.42-fold.

The increase in the average stress on the lateral condylar cartilage for NTEX was 1.80-fold, VAEX increased by 1.28-fold, and VGEX increased only by 1.11-fold.

### Meniscus (Fig. 5)

**Fig. 5:**
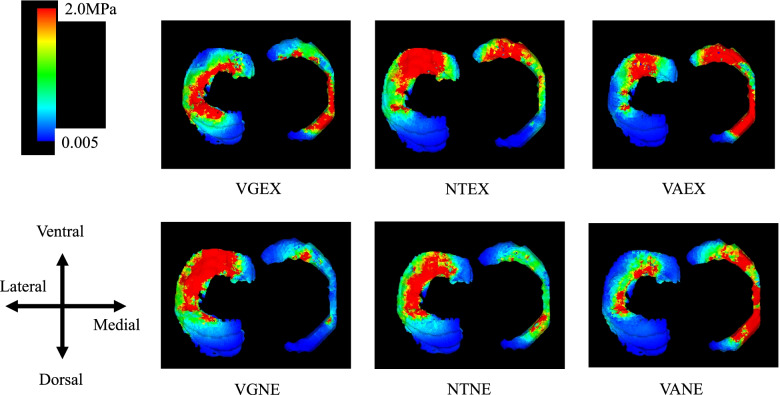
Axial view of meniscus showing von Mises stress. The von Mises stress was most concentrated in the medial meniscus in the VAEX model, followed by the VANE. VGNE; valgus and non-extrusion of medial meniscus, VGEX; valgus and extrusion of medial meniscus; NTNE; neutral and non-extrusion of medial meniscus, NTEX; neutral and extrusion of medial meniscus, VANE; varus and non-extrusion of medial meniscus, VAEX; varus and extrusion of medial meniscus

#### NTNE versus VANE and VGNE

The average stress on the medial condylar cartilage for VANE increased by 2.55-fold. On the other hand, for VGNE the stress increased by 0.52-fold. The average stress on the lateral condylar cartilage for VANE increased by 0.54-fold. On the other hand, for VGNE it increased by 1.21-fold.

#### NTNE versus NTEX, VAEX, and VGEX

The average stress on the medial meniscus for NTEX increased by 1.93-fold, and VAEX increased by 3.61-fold, but VGEX increased only by 1.65-fold. The average stress on the lateral meniscus for NTEX was 1.38-fold, for VAEX average stress also increased by 0.79-fold, and for VGEX it increased only by 1.24-fold.

**Fig. 6:**
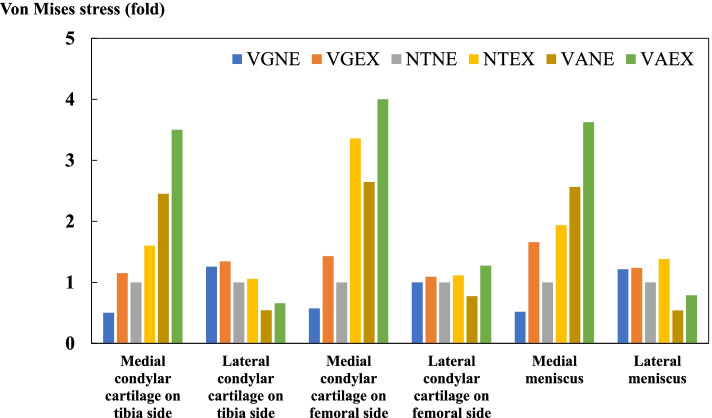
The mean of the von Mises stress on the condylar cartilage and meniscus, compared with that in an intact model. It was recognized that the tendency for von Mises stress at the medial cartilage and meniscus was increased in the VAEX model, however, the stress applied to the medial compartment in the VGEX model was almost the same as in NTNE. VGNE; valgus and non-extrusion of medial meniscus, VGEX; valgus and extrusion of medial meniscus; NTNE; neutral and non-extrusion of medial meniscus, NTEX; neutral and extrusion of medial meniscus, VANE; varus and non-extrusion of medial meniscus, VAEX; varus and extrusion of medial meniscus

It was noted that the von Mises stresses on the medial cartilage of tibial sides, as well as that on the meniscus were increased the most in the VAEX model, followed by the VANE model. On the femoral side, however, the stress increased most in the VAEX model, followed by the NEEX model. The von Mises stresses on the lateral cartilage of femoral and tibial sides and meniscus were comparable increased in both VGNE and VGEX models (Fig. 6).

## Discussion

### Stress changes in the medial compartment

This study revealed that the MME is associated with increased stress loading on the medial compartment structures (femoral and tibial cartilage and meniscus), and this pressure was enhanced by the larger JLCA (VAEX compared with NTNE; medial compartment: femoral 3.96- fold, tibia 3.52-fold, meniscus 3.61-fold).

Interestingly, In terms of stresses on the medial compartment of the knee joint, there was no difference in the effect of alignment between the femoral and tibial sides and the meniscus (VANE compared with NTNE; medial compartment: femoral 2.62-fold, tibia 2.49-fold, meniscus 2.55-fold), while the effect of MME was greater on the femoral side (NTEX compared with NTNE; medial compartment: femoral 3.34-fold, tibia 1.60-fold, meniscus 1.93-fold). MME was found in 50% to 100% of patients with SONK, with the extent of medial meniscus extrusion correlating to the stage and volume of SONK lesions [3, 23]. Although SONK occurs in both compartments of the femur or tibia, most of it occurs in the medial femoral condyle [24]. Based on the results of the present study, it is presumed that a large stress concentration may occur in the medial femoral condyle fastest when MME occurs.

Gokkus et al. analyzed the changes in stress distribution in the medial knee compartment depending on the different degrees of MME using the FE method [10]. According to that report, the contact pressure on the medial compartment of the knee joint increased linearly as the degree of MME increased. Furumatsu et al. also reported that the amount of MME increases over time [8]. The causality of lower leg alignment to MME remains controversial. Crema et al. reported that varus malalignment, meniscal tears, and cartilage damage were associated with MME [5] and Goto et al. reported a correlation between the degree of MME and knee varus alignment [11]. By contrast, Erquicia et al. did not find any association between varus malalignment and meniscus extrusion [7]. In actual clinical practice, there are some cases where MME is present but alignment is neutral, or cases where the JLCA is tilted to valgus direction in early knee OA patients.

As mentioned above, there are some cases in which the clinical symptoms are not severe and arthropathy changes do not progress much, even in the presence of MME [15]. In the present study, the contact pressure on the medial compartment in the condition with valgus alignment of JLCA did not increase significantly compared to that in the control model, even if the medial meniscus was extruded (VGEX compared with NTNE; medial compartment: femoral 1.42-fold, tibia 1.17-fold, meniscus 1.65-fold). Considering from the results of the present study, patients who originally had valgus alignment of the JLCA, even if MME was occurred, may not have severe symptoms and may not progress to arthritic changes.

Based on the results of this study, the JLCA should be considered in the treatment of patients undergoing arthroscopic surgeries such as pull-out repair or centralization.

### Stress changes in the lateral compartment

In examining the contact pressure on the lateral compartment, we found that MME increased the stress here as well as in the medial compartment (NTEX compared with NTNE; lateral compartment: femoral 1.80-fold, tibia 1.05-fold, meniscus 1.38-fold). Similar results have been reported in a biomechanical study of knee stress changes induced by MME that is caused by MMPRT in a cadaver knee [2]. Specifically, in the condition of MME applied to valgus alignment, the contact pressure of lateral tibial cartilage reached 2.36-hold compared with that in the control (VGEX compared with NTNE; lateral compartment: femoral 1.11-fold, tibia 2.36-fold, meniscus 1.24-fold). This is considered a large increase compared to the increase of about 1.25-hold in the condition which the JLCA was tilted in the direction to valgus without MME (VGNE compared with NTNE; lateral compartment: femoral 1.00-fold, tibia 1.25-fold, meniscus 1.21-fold). Thus, MME in patients with the JLCA of valgus alignment may require attention to subsequent degeneration of the lateral tibial compartment.

### Limitations

This study had some limitations. First, the analysis was performed solely in the full-extension position. The change in the morphology of the femoral condyle during the knee flexion and extension movement and its translation movements during the movement are tolerated by the meniscus. In a case that causes MME, such as meniscus root tear, this tolerance disappears, eccentric movements occur in the medial compartment, and this may cause arthrosis as a cause independent of the increase in load distribution. Further, the contacts between the meniscus and the tibia were simulated as a complete fusion, but this does not reflect the variation in pressures investigated by this study. Knee joint degeneration could have been influenced by various factors, such as sex, body mass index, and coverage rate. Moreover, the knee joint has a broad variation in formation and degenerative change, but a single model was used to assess the impact of meniscus and alignment on the knee joint. Lastly, knee joint stress was assumed to be influenced by the hip joint, pelvis, and foot, but our model was constructed from the femur to the lower leg. This was because the model from the hip joint to the ankle joint was too large and could not be analyzed, but this model seemed to be acceptable, which was similar to that in a previous study [18, 21].

## Conclusion

In the condition of varus alignment of JLCA with the MME, while the contact pressure of the medial compartment significantly increased, the condition of the valgus alignment of JLCA with MME showed almost no increase in the contact pressure on the medial compartment in the simulation using the FE method. Conversely, under these conditions, the pressure in the lateral compartment did increase. Thus, it might be necessary to determine treatment strategies based on the relationship between MME and JLCA in actual clinical practice.

NTNE implied neutral and non-extrusion of medial meniscus, NTEX was neutral and extrusion of medial meniscus, VANE was directed to varus and non-extrusion of medial meniscus, VAEX was directed to varus and extrusion of medial meniscus, VGNE was directed to valgus and non-extrusion of medial meniscus, and VGEX was directed to valgus and extrusion of medial meniscus.

## Data Availability

The datasets during and/or analyzed during the current study are available from the corresponding author on reasonable request.

## Abbreviations

OA: Osteoarthritis
ROAD: Research on Osteoarthritis/Osteoporosis Against Disability
ECM: Extracellular matrix
MME: Medial meniscus extrusion
SONK: Spontaneous osteonecrosis of the knee
MMPRT: Medial meniscus posterior root tear
FE: Finite element
CT: Computed tomography
KL: Kellgren-Lawrence
FTA: Femolo-tibial angle
MPTA: Medial proximal tibial angle
LDFA: Lateral distal femur angle
JLCA: Joint line convergence angle
DICOM: Digital imaging and communication in medicine
